# Anticoagulation for Left Ventricle Thrombus—Case Series and Literature Review for Use of Direct Oral Anticoagulants

**DOI:** 10.3390/jcdd10020041

**Published:** 2023-01-23

**Authors:** Akshyaya Pradhan, Monika Bhandari, Pravesh Vishwakarma, Chiara Salimei, Ferdinando Iellamo, Rishi Sethi, Marco Alfonso Perrone

**Affiliations:** 1 Department of Cardiology, King George’s Medical University, Lucknow 226003, India; 2 Department of Cardiology and CardioLab, University of Rome Tor Vergata, 00133 Rome, Italy

**Keywords:** left ventricular thrombus, anticoagulation, anterior wall myocardial infarction, dual therapy, direct oral anticoagulants

## Abstract

Left ventricular thrombus is a known complication following acute myocardial infarction that can lead to systemic thromboembolism. To obviate the risk of thromboembolism, the patient needs anticoagulation in addition to dual antiplatelet therapy. However, combining antiplatelets with anticoagulants substantially increases the bleeding risk. Traditionally, vitamin K antagonists (VKAs) have been the sheet anchor for anticoagulation in this scenario. The use of direct oral anticoagulants has significantly attenuated the bleeding risk associated with anticoagulation for atrial fibrillation and venous thromboembolism. Furthermore, in patients with atrial fibrillation undergoing percutaneous coronary intervention, the use of direct oral anticoagulants (DOACs) in conjunction with antiplatelets has been found to be noninferior in reducing ischemic events while significantly attenuating the bleeding compared with VKA. After initial case reports, multiple observational and nonrandomized studies have now safely and effectively utilized direct oral anticoagulants for anticoagulation in left ventricular thrombus. Here, we report a series of two cases presenting with left ventricular thrombus following acute myocardial infarction. In this case series, we try to address the issues concerning the choice and duration of anticoagulation in the case of postinfarct left ventricular thrombus. Pending the results of large randomized control trials, the judicious use of direct oral anticoagulant is warranted when taking into consideration the ischemic and bleeding profile in an individualized approach.

## 1. Introduction

A left ventricular (LV) thrombus is a known complication following acute myocardial infarction (AMI) that can lead to systemic thromboembolism. With the increasing use of timely thrombolysis and primary percutaneous interventions (PCIs), along with the unabated use of secondary prevention medications, the complications following AMI are decreasing and survival is improving [1]. After myocardial infarction (MI), LV thrombus still remains as high as 15% in the PCI era [2,3]. An LV thrombus usually occurs within 1 month post ST elevation MI, mostly occurs in the setting of acute anterior wall MI, and is associated with poor outcomes. The consideration of optimal anticoagulation, along with the decision of revascularization, makes decision-making a challenge. Echocardiography is the standard screening tool for detecting a thrombus, but sometimes contrast echocardiography might be required for confirming the diagnosis. The American College of Cardiology (ACC)/American Heart Association (AHA) guidelines for the management of AMI recommend oral anticoagulants (OAC) in addition to dual antiplatelet (DAPT) agents for the treatment and prevention of LV thrombi in acute MI [4]. However, the use of triple therapy comes at the cost of increased bleeding complications [5]. Bleeding following anticoagulation is also associated with an increase in mortality. Hence, balancing the ischemic benefits against bleeding events is a common clinical dilemma. The introduction of direct oral anticoagulants (DOACs) has revolutionized the scenario of the anticoagulation of vascular thromboembolism, including atrial fibrillation (AF). Studies conducted to assess the efficacy of dual therapy (single antiplatelet with OAC) in patients with acute coronary syndrome (ACS) and atrial fibrillation (AF) undergoing PCI have shown encouraging results with respect to attenuated bleeding and preserved efficacy. Additionally, DOACs have been found to be comparable with vitamin K antagonists (VKAs) [6,7,8,9]. Although these studies did not involve patients with an LV thrombus per se, a large body of affirmative data in the form of case reports, case series, observational studies and small randomized studies has emerged regarding the safety and efficacy of DOACs in treating LV thrombus. In this case series, we try to address this fairly common yet underestimated and underrepresented situation.

## 2. Case Summary

### 2.1. Case 1

**Figure 1:**
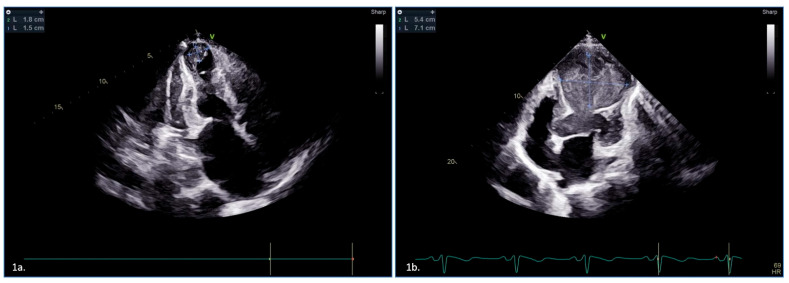
Echocardiographic demonstration of thrombus in two cases managed by different anticoagulation regimens. (**1a**)—two-dimensional echocardiography in apical view showing homogenous echo dense mass (1.8 × 1.5 cm) at apex of left ventricle, suggestive of thrombus. (**1b**)—two-dimensional echocardiography in apical view showing large echo dense mass (5.4 × 7.1 cm) at apex of a dilated and akinetic left ventricle, suggestive of thrombus.

A 46-year-old man with conventional cardiovascular risk factors presented with complaints of severe sudden onset chest pain of a 4-day duration. On examination, he had a dyskinetic apex with an LV third heart sound. His electrocardiogram was suggestive of anterior wall ST elevation MI, and his echocardiography showed a 1.8 cm × 1.5 cm clot at the apex (Figure 1a) and attendant severe LV dysfunction. The patient underwent coronary angiography, which revealed the 95% stenosis of the proximal left anterior descending artery with poor contractility of LV. In view of his severe LV dysfunction, late presentation, and pain-free status, he was subjected to myocardial perfusion imaging. Anticoagulation with VKA was initiated and was targeted to an INR 2.0–3.0. DOACs were not used, because the patient refused owing to financial constraints. Stress imaging (Technetium-99 single-photon emission computerized tomography) did not reveal any evidence of viability, and dual therapy was continued for 1 month. The LV thrombus resolved by the end of 1 month, but we still continued dual therapy (clopidogrel and oral warfarin), along with optimal medical treatment. He is planned for repeat echocardiography after 3 months and is under follow-up.

### 2.2. Case 2

A 66-year-old man presented to the emergency department on seventh-day post anterior wall ST elevation myocardial infarction. He was a known hypertensive and had an episode of ischemic stroke 3 years back. His echocardiography revealed a 5.4 cm × 7.1 cm LV thrombus at the apex with a cavity (Figure 1b). Because of the very late presentation, he was offered an option of ischemia-guided or symptom-guided revascularization. The patient opted for medical management and dual therapy with rivaroxaban, and clopidogrel was initiated at discharge. Interestingly, this thrombus revealed early partial resolution with dual therapy at the end of 1 month, and dual therapy was continued till 3 months. The repeat echocardiographic evaluation at 3 months failed to demonstrate any evidence of residual thrombus. He is presently on dual antiplatelet therapy and is doing well on follow-up.

## 3. LV Thrombus—An Overview

LV thrombus usually forms in the akinetic or dyskinetic segments of the ventricle post myocardial infarction (MI). The stasis of blood is maximal in these areas. Additionally, MI causes damage to the endocardium, and post MI, there is hypercoagulability; hence, according to the Virchow’s triad, these areas are prone to developing an LV thrombus. The incidence of LV thrombus post MI has been estimated to be between 15% and 25% in anterior wall MI by using cardiac MRI (magnetic resonant imaging) [3]. The incidence of LV thrombi is decreasing thanks to the increasing use of primary PCI, neurohormonal antagonists, adverse LV remodeling preventing agents and potent antithrombotic regimens. A cardiac MRI (CMRI) is the most specific and sensitive modality, making it the gold standard for detection of LV thrombus. However, in view of its limited availability, an echocardiogram remains the diagnostic tool of choice.

A recent study by Maniwa et al. has shown the incidence of systemic embolization to be 16.3% overall and 2.9% in patients maintaining an adequate therapeutic range of anticoagulation [10]. In another study, acute ischemic stroke occurred in 11.8% of patients with an LV thrombus who received anticoagulation as compared with 44.1% in those who were not on anticoagulants [11]. The protrusion of thrombi into the cavity, non resolving thrombi, and recurrent thrombi were predictors of stroke.

Once an LV thrombus is detected, patients should be immediately started on anticoagulation. In this scenario, anticoagulation provides benefits with respect to systemic thromboembolism, whereas antiplatelets provide benefits regarding ischemic events. According to the 2013 ACC/AHA ST elevation MI (STEMI) guidelines, it is reasonable to add OACs to DAPTs for patients with a STEMI and an asymptomatic LV thrombus, for 3 months, targeting a lower international normalized ratio (INR) goal of 2.0 to 2.5 [4]. On the other hand, the 2014 AHA/American Stroke Association (ASA) stroke prevention guidelines recommend anticoagulation for a similar duration, but with an INR target of 2.5 [12]. The 2017 European Society of Cardiology (ESC)’s STEMI guidelines recommend OACs for at least 6 months if there is an LV thrombus [13]. After 6 months, OACs are to be guided by repeated echocardiography and balancing bleeding risk and the need for concomitant antiplatelet therapy. However, these guidelines are not based on any randomized prospective studies in this scenario (AMI with an LV thrombus).

The guidelines recommend VKAs in the setting of LV thrombus because of more clinical experience. However, the need for frequent monitoring using the international normalized ratio (INR), food–drug interactions, and an inability to achieve the target therapeutic rate (TTR) are the major limitations of VKAs. Several observational studies and case reports have been conducted in this regard.

## 4. Clinical Experience of Combination of OACs with Antiplatelets

**Figure 2:**
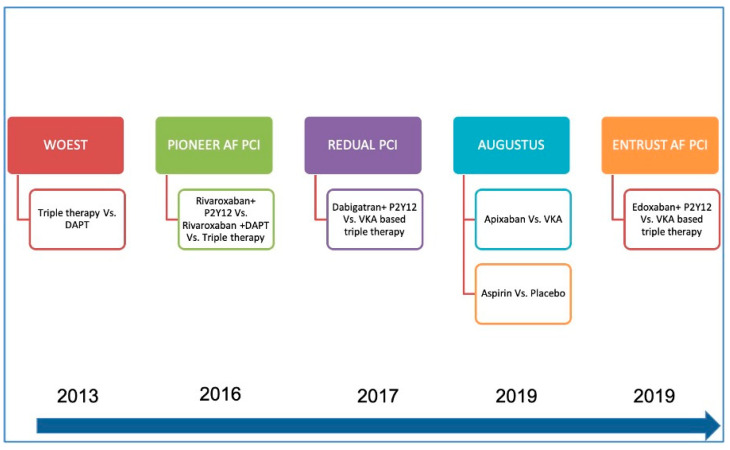
Timeline of pivotal trials comparing conventional triple therapy with dual therapy (either VKA or NOAC based). In the PIONEER AF PCI study, two doses of rivaroxaban were tried—2.5 mg and 5 mg. Similarly, in REDUAL PCI, both 110 mg and 150 mg doses were studied. AUGUSTUS PCI was a 2 × 2 factorial evaluating apixaban versus warfarin and aspirin versus no aspirin. (DAPT—dual antiplatelet therapy; P2Y12—clopidogrel; VKA—vitamin K antagonist).

It is well known that combining OACs with DAPTs substantially increases bleeding risk [5,14]. Triple therapy, however, may be initially considered in patients with high ischemic risk (recurrent MI, a suboptimal stent placement, or a history of stent thrombosis) [12]. While there are no studies that have compared dual therapy with triple therapy in the setting of MI and an LV thrombus, indirect evidence for the safety and efficacy of DOACs plus dual antiplatelets comes from trials of AF patients undergoing PCI (Figure 2 and Table 1) [15]. The WOEST and the ISAR-triple were the initial trials that included patients of AMI with AF requiring PCI that compared triple therapy against dual therapy with VKA [16,17]. In the WOEST trial, there was a significant reduction in serious bleeding (44% vs. 19.1%), and in the ISAR-triple trial, the shortening of clopidogrel therapy’s duration from 6 months to 6 weeks was found to be noninferior for both ischemic and bleeding events. Interestingly, the WOEST trial included patients not only with AF but also with other indications for anticoagulation. The four pivotal randomized trials of DOACs in patients of AMI with AF who underwent PCI have also shown the benefits of dual therapy (SAPT with DOACs) in reducing bleeding events primarily compared with VKA-based dual or triple therapy [6,7,8,9]. There were no differences in the ischemic events with DOAC-based therapy, though most of these studies were not powered enough for the detection of ischemic end points.

Meta-analyses of DOAC-based dual therapy have clearly shown that, compared with triple therapy (OACs with DAPT), their utilization leads to a marked decline in bleeding episodes without any increase in ischemic events. A meta-analysis of pivotal RCTs, by Lopes et al., revealed that the odds ratios (ORs) for TIMI major bleeding were 0.58 (95% CI, 0.31–1.08) for VKAs plus a P2Y12 regime, 0.49 (95% CI, 0.30–0.82) for DOACs plus a P2Y12 inhibitor regime, and 0.70 (95% CI, 0.38–1.23) for DOACs plus a DAPT regime, respectively, using VKAs plus a DAPT regime for comparison. Concurrently, the ORs for MACE were 0.96 (95% CI, 0.60–1.46) for VKAs plus a P2Y12 inhibitor, 1.02 (95% CI, 0.71–1.47) for DOACs plus a P2Y12 inhibitor, and 0.94 (95% CI, 0.60–1.45) for DOACs plus a DAPT, respectively [18]. The positive data from these studies set the stage for exploring novel oral anticoagulants (NOACs) in an LV thrombus in conjunction with antiplatelets.

## 5. DOACs in LV Thrombus—The Clinical Experience

### 5.1. Case Reports

The initial data emerged with multiple case reports that demonstrated a resolution of an LV thrombus with use of DOACs [19,20,21,22,23,24,25]. Most of these patients had an LV thrombus in the setting of acute MI. One of these cases was of hypertrophic cardiomyopathy, while two had nonischemic heart failure. The majority of patients had a resolution of the thrombus by the end of 1 month with DOAC use, while one case demonstrated thrombus resolution by as early as 7 days [24]. None of these reported any bleeding or systemic embolism with DOACs.

### 5.2. Observational Studies and Case Series

**Figure 3:**
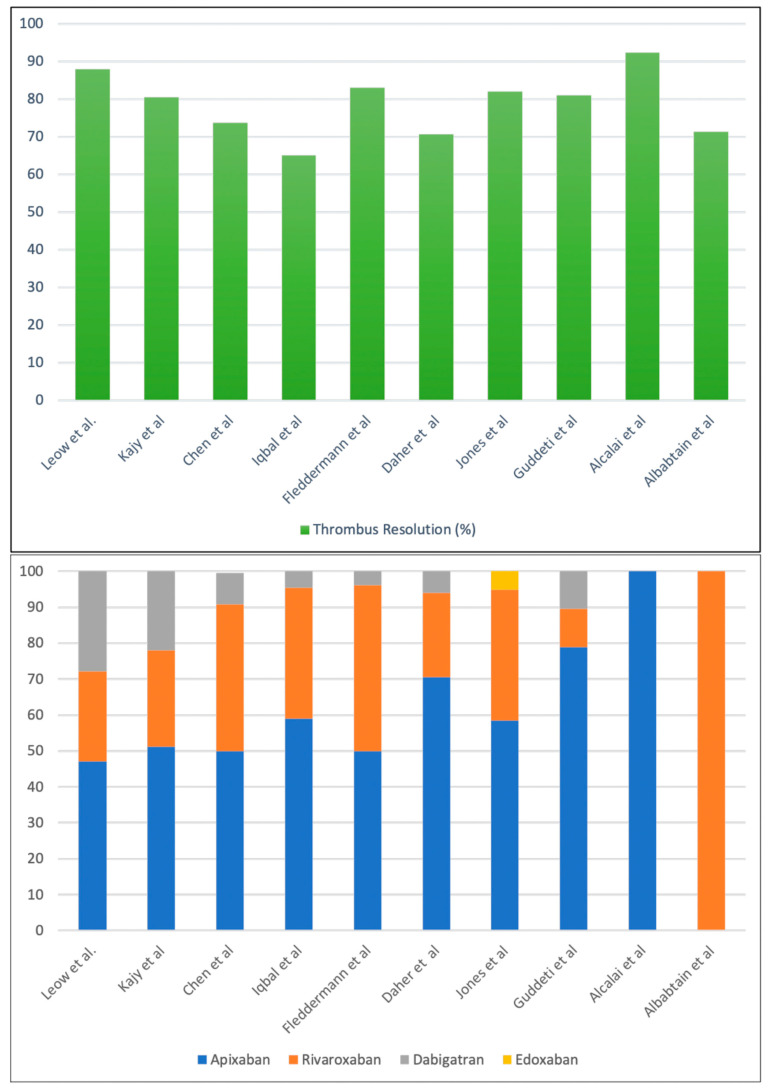
Rates of thrombus resolution observed in various studies/meta-analyses of studies utilizing DOACs for anticoagulation for LV thrombi (upper panel) and the proportion of various DOACs used in these studies (lower panel). Factor Xa inhibitor—apixaban has been the most extensively utilized DOAC, and edoxaban was the least favored. The first three columns represent meta-analyses, while the other seven represent individual studies.

Iqbal et al. performed a retrospective observational cohort study comparing DOAC therapy with VKAs in patients with an LV thrombus [26]. In this study, 74% patients received warfarin, and 26% patients received DOACs. There was no significant difference in the rate of stroke or that of other thromboembolic events between the groups (2% vs. 0%, respectively, *p* = 0.55). There were six episodes of clinically significant bleeding in the study, all of which were seen with warfarin-based triple therapy (10% vs. 0%, *p* = 0.13). The indication of fewer bleeding events with DOAC-based triple therapy as compared with VKA-based triple therapy was clearly apparent. Subsequently, multiple retrospective and prospective observational studies and case series have evaluated DOACs for anticoagulation in the context of an LV thrombus, as summarized in Table 2 [27,28,29,30,31,32,33,34,35]. Thrombus resolution on follow-up was the major end point in most of the studies, and DOACs were similar or superior to VKAs in all of them (Figure 3). Both the rate of resolution and the time of resolution were equal or better with DOACs. Bleeding events were similar or lower with DOACs in all the studies described in the Table when compared with VKAs. In one study, bleeding events necessitating transfusion were noted with DOACs, but all these patients had concomitant antiplatelets [29]. Additionally, systemic embolism and stroke rates were evaluated by many, and DOACs were again as efficacious as VKAs in the majority. Although inherently limited by their nonrandomized nature, the variability of the types, the doses of DOACs utilized, and the nonuniform end points evaluated, the plethora of studies do herald the era of DOAC anticoagulation for LV thrombi.

### 5.3. Randomized Controlled Trial Experience

As noted above, there is paucity of RCTs for NOAC use in LV thrombi. The recently published NO-LVT study is possibly the only RCT comparing rivaroxaban (20 mg OD) with warfarin [36]. The main outcome was thrombus resolution at 1, 3, and 6 months, assessed by echocardiography, while bleeding and systemic embolism were secondary end points. In the warfarin arm, enoxaparin bridging was employed until the INR reached 2–3. Of the 79 patients randomized, complete thrombus resolution was seen in 72%, 77%, and 87% at months 1, 3, and 6, respectively, with rivaroxaban. The corresponding figures with VKAs were 48%, 67%, and 80%. At 1 month, the odds of thrombus resolution were higher with rivaroxaban compared with VKAs (187 OR—2.8; *p* = 0.03). No embolic events (stroke or systemic embolism) were seen with rivaroxaban, while bleeding was numerically lower with rivaroxaban. The major limitation was the use of TTE for assessing LV thrombi, but that is the general practice worldwide. The trial comes as a shot in the arm for DOAC use in LV thrombi.

### 5.4. Meta-Analysis and Systematic Reviews

Many meta-analyses, meta-summaries, and systematic reviews based on these observational studies and case reports have also shown the equivalent efficacy and better safety of DOACs in treating patients with an LV thrombus (Table 3) [37,38,39,40,41,42,43,44,45].

One of the largest meta-analyses was conducted by Chen et al., which included 2467 patients on anticoagulation for an LV thrombus. The common theme again was the better efficacy of DOACs for thrombus resolution, with no difference in systemic embolism or stroke. The risk of bleeding was also found to be similar between VKAs and DOACs in most of these meta-analyses.

Three recent meta-analyses of anticoagulation in LV thrombi have also shown similar results. In a systemic meta-analysis conducted by Shu Fang et al., which included 2262 patients from 12 observational studies, there was no difference in safety or efficacy. The rates of systemic embolism and stroke were 18.8% for DOACs and 22.6% for VKAs, OR = 1.01, and 8.8% vs. 11.4%, OR = 0.76, respectively. Thrombus resolution also showed similar trends in two groups (80.6% for DOACs vs. 80.2% for VKAs). However, it was noted that there were fewer bleeding and systemic embolism episodes, although these were not statistically significant. Thus, DOACs might be a safer option [46].

Similar results were reported in a meta-analysis conducted by Tetsuji Ketano et al. comprising 2612 patients. There was no difference in thrombus resolution (0.75 for VKAs vs. 0.75 for DOACs), stroke (0.06 for VKAs vs. 0.02 for DOACs), or any embolism (0.08 for VKAs vs. 0.03 for DOACs. The odds ratio for major bleeding was 0.06 for VKAs vs. 0.03 for DOACs [47].

Another meta-analysis, conducted by H. da Silva Ferraira, showed almost-equivalent efficacy and safety for both types of OACs (stroke/systemic embolism for DOACs: 109/618 vs. 386/1814 for VKAs (OR 0.86; 95% CI, 0.55–1.33; *p* = 0.50); any bleeding event: 8.7% of DOAC patients and 8.3% of VKA patients (OR 0.96, 95% CI 0.62–1.48, *p* = 0.88)) [48].

Thus, it can be concluded from these meta-analyses that DOACs are noninferior to VKAs in terms of thrombus resolution, with no difference in the risk of stroke or embolism.

**Table 3:** Meta-analyses, meta-summaries, and systematic reviews comparing DOAC therapy and VKA therapy in setting of LV thrombus following MI. Notes: IQR—interquartile range; DOAC—direct oral anticoagulant; OAC—oral anticoagulant; VKA—vitamin K antagonist; LV—left ventricle; OR—odds ratio.

Author (Year)	Sample Size	Study Drug	End Points	Results	Safety
Leow et al. (2018) [37]	36	Rivaroxaban (47.2%) Apixaban (25.0%) Dabigatran (27.8%)	Thrombus resolution and time to resolution.	Thrombus resolution was observed in 87.9%, and median duration of treatment to resolution was 30.0 days (IQR = 22.5–47.0).	1 nonfatal bleeding event (3.0%); no embolic events.
Kajy et al. (2020) [38]	41	Rivaroxaban (51.2%) Apixaban (26.8%) Dabigatran (22%)	Thrombus resolution and time to resolution.	Thrombus resolution—81% (R), 100% (A), and 88.9% (D). Median time of resolution—40 days (R), 36 days (A), and 24 days (D).	One nonfatal bleeding event and one stroke event were reported while on a DOAC.
Al-abcha et al. (2020) [39]	857	VKAs = 480; DOACs = 220	Primary outcome was thrombus resolution, and the secondary outcomes were major bleeding and stroke or systemic embolism (SSE).	Similar rate of thrombus resolution (odds ratio (OR) 0.97; *p* = 0.90).	Major bleeding (OR 0.62; *p* = 0.27) and systemic embolism (OR 1.86; *p* = 0.05) were not different between groups.
Chen et al. (2021) [40]	2467	Among DOAC users, apixaban (50.0%), rivaroxaban (40.8%), dabigatran (8.8%), and edoxaban (0.4%); among VKA warfarin (98.5%) was predominantly prescribed	Stroke or systemic embolism; thrombus resolution.	For prevention of stroke or systemic embolism (VKA vs. DOAC—RR: 0.96, 95% confidence interval (CI): 0.80–1.16, *p* = 0.68); for thrombus resolution (VKA vs. NOAC—RR: 0.88, 95% CI: 0.72–1.09, *p* = 0.26); for risk of stroke (VKA vs. DOAC—RR: 0.68, 95% CI: 0.47–1.00, *p* = 0.048).	For risk of any bleeding; no difference between VKAs and DOACs (RR: 0.94, 95% CI: 0.67–1.31, *p* = 0.70); for clinically relevant bleedings—lower risk with DOAC users (RR: 0.35, 95% CI: 0.13–0.92, *p* = 0.03) compared with VKA users.
Burmister et al. (2021) [41]	2153	570 on DOACs vs. 1583 on VKAs)	LV thrombus resolution, thromboembolic events, and thromboembolic stroke.	Thrombus resolution was significantly higher in DOACs compared with VKAs (RR: 1.18 (95% CI: 1.04–1.35); *p* = 0.01, I2 = 25%); no significant difference existed between DOACs and VKAs regarding overall thromboembolic events (RR: 1.10 (95% CI: 0.75–1.62); *p* = 0.61) or embolic strokes (RR: 0.63 (95% CI: 0.39–1.02); *p* = 0.06).	No difference in all-cause death (RR-0.84, *p* = 0.53) or bleeding (RR-1.00, *p* = 0.9).
Trongtorsak (2021) [42]	1771	DOACs—426 and VKAs—1345	Stroke, systemic embolism. Thrombus resolution, bleeding.	No significant differences in rates of systemic embolism or LV thrombus resolution.	Bleeding similar between two groups.
Shah et al. (2021) [43]	867		Systemic embolism and LV thrombus resolution.	Systemic embolic events (SEE)—2.7%; thrombus—86.6%.	Bleeding (composite of major and minor) and major bleeding—5.6% and 1.1%, respectively.
Abdelaziz et al. (2021) [44]	700	VKAs = 480; DOACs = 220.	Stroke or systemic embolism (SSE). Secondary outcomes were thrombus resolution, bleeding, and death.	For stroke or systemic embolism (SSE), lower rates with VKAs compared with DOACs (5.2% vs. 9%; OR = 0.54, *p* = 0.05).	Rates of thrombus resolution (OR = 1.00, *p* = 0.99) and bleeding (OR = 1.62, *p* = 0.27) and death (OR = 1.09, *p* = 0.79) were similar.
Tetsuji Ketano et al. (2021) [47]	2612	VKAs = 2004; DOACs = 608	Thrombus resolution, stroke, any thromboembolism, and major bleeding.	No difference in thrombus resolution (0.75 for VKAs vs. 0.75 for DOACs), stroke (0.06 for VKAs vs. 0.02 for DOACs), or any embolism (0.08 for VKAs vs. 0.03 for DOACs).	OR for major bleeding -0.06 & 0.03 for VKAs DOAC respectively.
Saleh et al. (2021) [45]	2395		Primary—thrombus resolution; secondary—occurrence of major bleeding and stroke or systemic embolization (SSE).	The rates of thrombus resolution for VKAs and DOACs were equal (71.9% vs. 71.4%; *p* = 0.36). Systemic embolism was also similar between arms (21.3% and 15.6%, respectively; *p* = 0.57).	Major bleeding rates were similar between DOACs and VKAs. (8.2% vs. 7.1%, *p* = 0.57, OR = 0.87).
Shu Fang et al. (2022) [46]	2262	VKAs = 1575; NOACs = 570	Thrombus resolution, stroke/SSE, bleeding, and mortality.	The rate for SSE (OR 1.01, *p* = 0.95) and that for thrombus resolution (OR = 1.15) were similar.	Similar bleeding risk(OR = 0.78).
H. da Silva Ferraira (2022) [48]	2432	DOACs = 618; NOACs = 1814	Stroke/SSE and bleeding events.	DOACs vs. VKAs (OR = 0.86).	8.7% for DOACs vs. 8.3% for VKAs.

### 5.5. Use of Anticoagulation for Prevention of LV Thrombus Formation

Previously, conventional triple therapy comprising DAPT plus VKAs was used to prevent LV thrombus formation in patients with a high-risk ST elevation MI such as large anterior wall ST elevation MI—LV ejection fraction < 30%, dyskinetic LV, or formation of LV aneurysm [46]. However, this practice was not supported by high-quality evidence. Moreover, triple therapy increased the risk of major bleeding, so interest in this area has been waning.

Zhang et al. studied the prophylactic use of rivaroxaban for LV thrombus after anterior ST elevation MI [49]. The study comprised 279 patients who underwent PCI and were randomized in a one-to-one manner to either rivaroxaban (2.5 mg twice daily for 30 days) plus DAPT or DAPT alone. The primary end point was the formation of an LV thrombus within 30 days. The net clinical adverse event included all-cause mortality, LV thrombus formation, systemic embolism, rehospitalization for cardiovascular events, and bleeding. There was a significant reduction in LV thrombus formation by rivaroxaban (0.7% vs. 8.6%). The net adverse events were also lower in the rivaroxaban group, while there were no differences in bleeding events at 30 or 180 days.

Thus, the use of the shortest possible course of triple therapy comprising DOACs with the further continuation of DAPT as required may be used for at-risk patients and is an area of research in such patients. However, further research is needed in this regard for strong validation [49].

### 5.6. A Note of Dissent

While the majority of case reports, case series, and nonrandomized studies favor the use of DOACs in settings involving an LV thrombus, some have produced disparate results. Robinson et al. demonstrated higher stroke and systemic embolism rates (hazard ratio: 2.6–2.7) with DOAC use for an LV thrombus [34]. The large sample size, multicenter design, and longer follow-up are strengths of the study. Similarly, Abdelaziz, in a recent meta-analysis, found that lower rates of stroke and systemic embolism were noted with VKAs vs. DOACs [43]. These results advocate caution and argue against the blanket use of DOACs for LV thrombi without additional consideration of ischemic and bleeding risks.

The genesis of LV thrombi is multifactorial, including stasis and endothelial dysfunction, whereas the left atrial (LA) thrombus is primarily stasis induced. It also noteworthy that DOACs in AF are used principally to prevent the genesis of an LA thrombus, but the thrombus is already in situ in the current scenario. In this case, the type of DOAC could be of importance: factor Xa inhibitors versus direct thrombin inhibitors. One hypothesis postulated is that dabigatran binds thrombin in a one-to-one molecular ratio, while one factor Xa leads to the generation of 1000 thrombin molecules, making factor Xa inhibition more attractive. Few cases of LV thrombi on dabigatran therapy have emerged in the literature [50,51]. The use of dabigatran for anticoagulation in mechanical heart valves was also unsuccessful in a RE-ALIGN study. However, factor Xa inhibitors have been preferentially utilized in LV thrombus studies (Figure 3).

Interestingly, Robinson et al. found no effect from oral/parenteral anticoagulation use on LV thrombus resolution during follow-up. This contrasts with previous studies and conventional wisdom. Contemporary studies have shown that prolonged anticoagulation attenuates rates of major adverse cardiovascular events and embolic events in patients with an LV thrombus [52].

An analogy can be drawn from the use of DOACs in situations including AF undergoing PCI. DOAC-based combination therapy has now shown to be noninferior in comparison to warfarin-based therapy in reducing ischemic events while showing simultaneous superiority in reducing serious bleeding in AF patients undergoing PCI [15]. However, none of the individual trials were powered enough to assess the ischemic events. In fact, some signals of numerically increased stent thrombosis have emerged in a meta-analysis, advising caution [53].

The use of an echocardiographic resolution of LV thrombi as an end point is marred by the low sensitivity of echocardiography, the varied time interval between echocardiographic acquisitions, and the differential frequency of imaging used in these studies, calling for clinical event-driven end points in future studies.

More recently, the failure of two large DOAC trials in rheumatic heart disease and prosthetic heart valves, respectively, further bolsters the role of VKAs as a first-line therapy for non-AF-based indications of OACs. Rivaroxaban failed to improve outcomes compared with VKAs in the large randomized INVICTUS study in the setting of rheumatic mitral valve disease [54]. Similarly, a trial of apixaban in the setting of prosthetic heart valves (the ON-X valve in the PROACT-Xa trial) was stopped prematurely owing to futility [55]. Though the results are not generalizable to the current context, they at least give an indication for slowing down the pace of the universal acceptance of DAOCs for LV thrombi.

## 6. Future Directions

Large and adequately powered RCTs comparing DOACs and VKAs with at least 6–12-month follow-ups are the need of the hour. With the prompt revascularization and institution of secondary prevention therapies attenuating the rates of LV thrombus formation following MI, this seems to be an uphill task. The **EARLYmyo-LVT (NCT03926780**; n = 280) is an ongoing study comparing rivaroxaban (15 mg OD) with warfarin (target INR: 2–2.5) as a part of triple therapy post MI. The rate of thrombus resolution at 3 months and bleeding events are the primary end points. Another ongoing study (**NCT03232398**) is comparing apixaban (5 mg BD) in LV thrombi versus warfarin (target INR: 2–3) for post MI. The primary end point again is the echocardiographic resolution of a thrombus after 3 months of therapy, and it plans to recruit 50 patients.

## 7. Choice for Anticoagulation—Practical Considerations and Guidelines

### 7.1. Utilizing Risk Scores for Decision-Making

The choice of DOACs versus VKAs for the anticoagulation regime is a matter of debate. In the absence of large RCTs, few practical considerations deserve merit. Three potential factors need to be considered: bleeding risk with VKAs (assessed by a **HAS-BLED** score), the ability to maintain therapeutic INR with VKAs (assessed by a **SAMeTT2R2** score), and financial considerations. If the patient has a high bleeding risk and/or there is difficulty in achieving therapeutic INR, DOACs should be preferred. Otherwise, VKAs should be the choice for anticoagulation. Additionally, when there are financial constraints, VKAs should be used thanks to their low cost. When combining OACs and DAPT, the duration of triple therapy should be kept to no longer than 1 month, according to the data extrapolated from trials of AF patients undergoing PCI [6,7,8,9,15].

A recently published review article on triple therapy in the setting of PCI suggested that therapy can be individualized on the basis of patients with thrombotic and bleeding risks, by taking into account the time frame post PCI. The authors suggested four time frames, 0–1 month, >1–6 months, >6–12 months, and >12 months. In the first month post PCI, all the patients can be given DOACs plus P2Y12 inhibitors, and aspirin can be added in those patients with high thrombotic but low bleeding risks. After 1 month and until 6 months, all patients are to be kept on DOACs plus P2Y12 inhibitors. In the next 6 months, patients with low bleeding risk to be kept on DOACs plus P2Y12 inhibitors, irrespective of thrombotic risk, and only on DOACs if the bleeding risk is high. Beyond 12 months, all the patients should be on DOACs only [56].

### 7.2. Suggested Algorithm

In medically managed patients, a dual therapy is preferred in order to curtail the bleeding risk while patients undergoing PCI will need an initial triple therapy regimen (DAPT+OAC). For one of our patients, we prescribed dual therapy with VKAs, while for another patient, we gave dual therapy with DOACs. Interestingly, both patients responded well to dual therapy, and there was a resolution of the LV thrombus at 1 month. More importantly, there were no thromboembolic events; neither were there any bleeding episodes. Certain clinical features that predict a high risk of stroke, such as a prior systemic embolism, the protrusion of a thrombus into the cavity, a recurrent thrombus, and the nonresolution of a thrombus from the initial therapy, may call for the preferential use of warfarin-based anticoagulation [10,34]. Patients with a high risk of stent thrombosis (recurrent ACS, multiple stents, complex bifurcation PCI, heavily calcified lesions, total stent length >60 mm, or bioabsorbable stents) may benefit from the extended duration of initial triple therapy [57].

**Figure 4:**
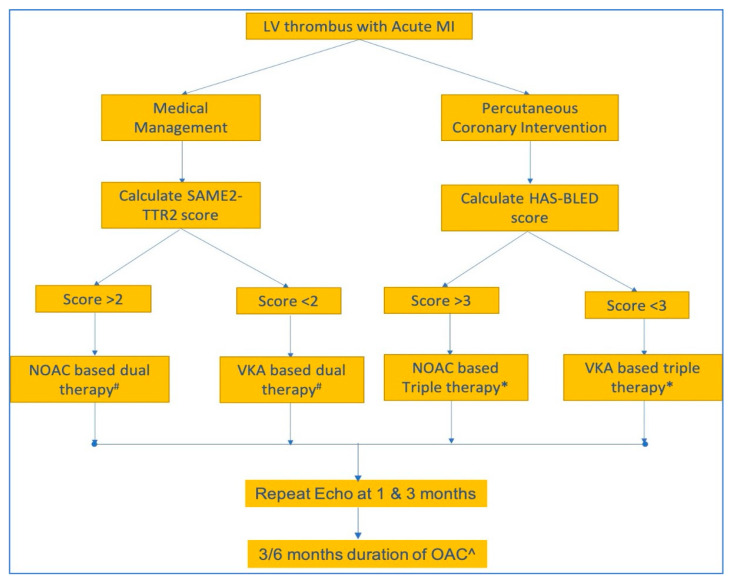
An approach to anticoagulation in LV thrombus complicating acute MI utilizing risk stratification scores. Dual therapy refers to a combination of single antiplatelet agent and oral anticoagulation. Triple therapy refers to a combination of dual antiplatelet therapy and oral anticoagulation. Presence of high-risk features warrants upgradation to VKAs from NOACs or triple therapy from dual. The variables used in HAS-BLED score include hypertension, abnormal renal or liver enzymes, stroke, bleeding events, labile INR, elderly age (>65 yrs), and drugs or ethanol. The SAMeT2TR2 score variables are sex (female), age (< 60 yrs), medical history, treatment strategy (rhythm control), tobacco use, and race (nonwhite race). Notes: #—prasugrel and ticagrelor should be avoided in dual or triple therapy; *—generally continued for 1 month, followed by dual therapy to avoid bleeding, and in the case of embolic events, it can be continued beyond 1 month; ^- ACC/AHA and CCS/CAIS guidelines advocate a 3-month regimen, while ESC guidelines prescribe a 6-month duration.

A suggested algorithm regarding the choice and duration of anticoagulation use in LV thrombi that is based on the current literature is presented in Figure 4.

Nonetheless, the lack of a predictable anticoagulant response, narrow therapeutic range, and need for frequent monitoring has spurred the more widespread use of DOACs and use of DOAC-based combination therapy in AMI patients who require concomitant oral anticoagulation. Because rivaroxaban and apixaban are now off patent, the financial constraints may no longer be a valid argument in many geographical regions, leading to increased prescriptions. However, as previously detailed, there is no need to jump the queue in utilizing DOACs until their noninferiority is established in large RCTs, and they should still be alternatives to VKAs on case-by-case bases.

### 7.3. Guideline Track

The 2014 ASA guidelines do recommend the use of DOACs in patients who are intolerant to warfarin [12]. In patients with apical akinesis/dyskinesis, OAC use has been given a Class IIb recommendation by the 2013 ACC/AHA guidelines for the management of a STEMI, as well as by the 2014 ACC/ASA guidelines for the prevention of stroke [4,12]. The 2018 Canadian Cardiovascular Society/Canadian Association of Interventional Cardiology Guidelines recommend only VKAs for patients with an established LV thrombus undergoing PCI for acute or stable indication [57]. They suggest the discontinuation of OACs beyond 3 months if there is no echocardiographic evidence of a thrombus, similar to ACC/AHA guidelines. They do acknowledge a lack of adequate evidence in this scenario.

The recent scientific statement of the AHA on the management of LV thrombi suggests using anticoagulation for 3 months, and thereafter, imaging should be performed to determine thrombus resolution. For patients with a history of a more distant MI, a longer duration of OACs up to 6 months may be considered. If there is a resolution of the LV thrombus, anticoagulation can be stopped. However, in case imaging is needed before 3 months for some other reasons and there is thrombus resolution, anticoagulation can be stopped earlier. It also suggests that if there is a clinical suggestion of a LV thrombus and the echo does not visualize a thrombus or if the echo is not confirmative, a cardiac MRI (CMR) should be conducted. It also suggests DOACs as reasonable alternatives to warfarin on the basis of supportive evidence. If the thrombus persists beyond 3 months, particularly a protruding thrombus, a trial of alternative anticoagulation should be considered: the use of DOACs with repetitive subtherapeutic INR if the patient was on warfarin or the use of warfarin if the patient was previously on DOACs [58].

Because of the relatively weak evidence, these latest guidelines suggest that the use of OACs in patients with revascularized anterior MI (usually primary PCI) may be considered. However, such a consideration should take into account the perceived risk of thrombus formation and bleeding risk and should involve shared decision-making. The treatment duration should be 1–3 months, depending on the bleeding risk [58].

Additional maneuvers that can be utilized to attenuate bleeding risk while combining antiplatelets with antithrombotic are summarized in Table 4.

## 8. Conclusions

According to the current evidence, it can be stated that if an LV thrombus is detected in a setting of AMI, (VKA-based) oral anticoagulation targeted to an INR of 2.0–3.0 has been the standard of care. DOACs have emerged as acceptable alternatives to VKAs in this scenario, owing to challenges in their use—such as their high bleeding risk, food interactions, need for repeated INR monitoring, and failure to achieve therapeutic range in many patients. A plethora of successful studies in the form of case reports, case series, observation studies, small RCT and meta-analyses have now demonstrated the utility of DOACs in better thrombus resolution and less bleeding. There have been some signals of an increased risk of stroke and systemic embolism in some studies with DOACs, but unfortunately, there have been no large randomized studies to date. Hence, if a patient is unable to achieve therapeutic INR (high SAME-TT2R2) or if they have a high bleeding risk (high HAS-BLED) with VKAs, full-dose DOACs should be prescribed instead of VKAs. This approach has the potential to attenuate bleeding risks while preserving efficacy [59]. The duration of anticoagulation is not defined but should be continued for at least 3 months, guided by a similar imaging modality to what was used earlier (or CMR if needed), to evaluate the resolution of an LV thrombus. If there is no LV thrombus on repeated echocardiographic evaluations, OACs can be stopped and DAPT should be started, which can be continued for 1 year. A repeat imaging after the cessation of OACs is prudent to detect the recurrence of a thrombus or a small nidus of a thrombus previously missed. In patients who continue to have some spontaneous echo contrast or a well-organized thrombus in the area of a wall motion abnormality, the optimal duration of OACs is not well defined. The further continuation of OACs can be made on a case-by-case basis. Large and well-designed trials comparing VKAs and DOACs in this setting are warranted.

## Figures and Tables

**Table 1:** Trials of oral anticoagulation therapy comparing dual therapy with triple therapy in patients with atrial fibrillation with acute coronary syndrome. Notes: DAPT—dual antiplatelet; ISTH- International Society of Thrombosis and Hemostasis; VKA—vitamin K antagonist; C—clopidogrel; A—aspirin; TIMI—thrombolysis in myocardial infarction.

Trial	Year	Drugs Compared	Number	Follow-Up	Primary End Points
WOEST	2013	VKA + C, VKA + DAPT	563	12 months	Total number of TIMI bleeding events
ISAR-TRIPLE	2015	VKA + A, VKA + DAPT	614	9 months	Composite of death, MI, definite stent thrombosis, stroke, and TIMI major bleeding
PIONEER- AF PCI	2016	Rivaroxaban (2.5/5) + C, VKA + DAPT	1415	12 months	A composite of major bleeding or minor bleeding event according to the TIMI or bleeding requiring medical attention.
REDUAL PCI	2017	Dabigatran (110/150) + C, VKA + DAPT	2725	24 months	A composite of major or clinically relevant nonmajor bleeding event according to ISTH
AUGUSTUS PCI	2019	VKA + C, Apixaban + C, VKA + DAPT	4614	6 months	A composite of major or clinically relevant nonmajor bleeding event according to ISTH
ENTRUST AF PCI	2019	Edoxaban + C, VKA + DAPT	1506	12 months	Major or clinically relevant nonmajor bleeding event according to ISTH

**Table 2:** Data from observational studies comparing anticoagulation with DOACs vs. VKAs in patients with LV thrombus. Notes: DOAC—direct oral anticoagulant; OAC—oral anticoagulant; VKA—vitamin K antagonist; LV—left ventricle; HR—hazard ratio.

Study	Number of Patients	Anticoagulant Profile	End Points	Follow-Up	Outcome
Robinson et al. (2018) [27]	84	No OAC: 16 patients Warfarin: 40 patients NOACs: 35 patients Other OACs: 7 patients	Survival free of stroke and systemic embolism	1 year	No difference 88% vs. 77.9%, *p* = 0.719.
Jaidka et al. (2018) [28]	49	Warfarin: 37 patients NOACS: 12 patients	Thrombus resolution, embolic events, bleeding events	6 months	No difference in bleeding or embolic events. Thrombus resolution also not different between VKAs and NOACs (69.2% vs. 88.9%; *p* = 0.245).
Fleddermann et al. (2019) [29]	52	Only NOAC—apixaban = 26 Rivaroxaban = 24 Dabigatran = 2	Rate of LV thrombus resolution; bleeding	264 days	83% had resolution of LV thrombus on follow-up echocardiogram. 1 cardioembolic event and 4 bleeding events requiring transfusion.
Daher et al. (2020) [30]	59	Warfarin: 42 patients NOACS: 17 patients	Rate of LV thrombus resolution	3 months	Thrombus resolution was similar in patients on NOACs (70.6%) and those on VKAs (71.4%; *p* = 0.9).
Jones et al. (2020) [31]	101	Warfarin: 60 patients NOACS: 41 patients	Primary—rate of LV thrombus resolution; secondary—rate of bleeding	2.2 years	Thrombus resolution earlier and greater with NOACs (82% vs. 64.4%, *p* = 0.0018). Bleeding rates lower with NOACs (0% vs. 6.7%, *p* = 0.030). No difference in rates of systemic thromboembolism (5% vs. 2.4%, *p* = 0.388).
Guddeti et al. (2020) [32]	99	Warfarin: 80 patients NOACS: 19 patients	Occurrence of ischemic stroke, bleeding, and thrombus resolution	1 year	No difference between stroke within 1 year or bleeding between two groups (numerically higher event in warfarin group); thrombus resolution was similar between groups (80% vs. 81%, *p* = 0.9).
Alcalai et al. (2020) [33]	25	Warfarin: 12 patients Apixaban: 13 patients	Primary end point: thrombus resolution Secondary end point: systemic embolism, major bleeding, and death from any cause	3 months	Complete thrombus resolution in all patients with warfarin and 12 out of 13 patients in apixaban group. 2 major bleeding events in warfarin group and none in apixaban group.
Robinson et al. (2020) [34]	514	Warfarin: 300 patients NOACs (apixaban in majority): 185 patients No OAC: 93 patients 64 switched regimens	Stroke and systemic embolism (SSE)	~1 year (351 days)	NOAC use associated with higher SSE risk compared with VKA use (HR—2.64–2.71); prior stroke or embolism also associated with higher SSE risk.
Albabtain et al. (2021) [35]	63	Warfarin: 35 patients NOAC (rivaroxaban): 28 patients	Time to thrombus resolution, bleeding, stroke, and mortality	9.5 months	Median time to thrombus resolution faster with NOACs (9 months vs. 3 months, *p* = 0.019); no difference in embolism, bleeding, or mortality.

**Table 4:** Methods to mitigate bleeding risk with a combination of antiplatelet and anticoagulant therapy.

Methods
Use of lower doses of aspirin
Proton pump inhibitor use
Avoid potent P2Y12 inhibitor—ticagrelor and prasugrel
Shorten the duration of DAPT
De-escalation of DAPT
Radial access in case of PCI
Sparing use of glycoprotein IIb/IIIa
